# A Narrative Review of Genetic Alterations in Primary Thyroid Epithelial Cancer

**DOI:** 10.3390/ijms22041726

**Published:** 2021-02-09

**Authors:** Cristina Romei, Rossella Elisei

**Affiliations:** Department of Clinical and Experimental Medicine, Section of Endocrinology, University of Pisa, 56124 Pisa, Italy; cristina.romei@unipi.it

**Keywords:** thyroid cancer, oncogenes, molecular signature, *RET*, *BRAF*, *TERT*, *p53*

## Abstract

Thyroid carcinoma is the most frequent endocrine neoplasia. Different types of thyroid carcinoma are described: well-differentiated papillary thyroid carcinoma (PTC), poorly differentiated thyroid carcinoma (PDTC), follicular thyroid carcinoma (FTC), anaplastic thyroid carcinoma (ATC), and medullary thyroid carcinoma (MTC). MTC is inherited as an autosomal dominant trait in 25% of cases. The genetic landscape of thyroid carcinoma has been largely deciphered. In PTC, genetic alterations have been found in about 95% of tumors: BRAF mutations and *RET* rearrangements are the main genetic alterations. BRAF and *RAS* mutations have been confirmed to play an important role also in PDTC and ATC, together with TP53 mutations that are fundamental in tumor progression. It has also been clearly demonstrated that telomerase reverse transcriptase (TERT) promoter mutations and TP53 mutations are present with a high-frequency in more advanced tumors, frequently associated with other mutations, and their presence, especially if simultaneous, is a signature of aggressiveness. In MTC, next-generation sequencing confirmed that mutations in the *RET* gene are the most common molecular events followed by *H-RAS* and *K-RAS* mutations. The comprehensive knowledge of the genetic events responsible for thyroid tumorigenesis is important to better predict the biological behavior and better plan the therapeutic strategy for specific treatment of the malignancy based on its molecular profile.

## 1. Introduction

**Figure 1:**
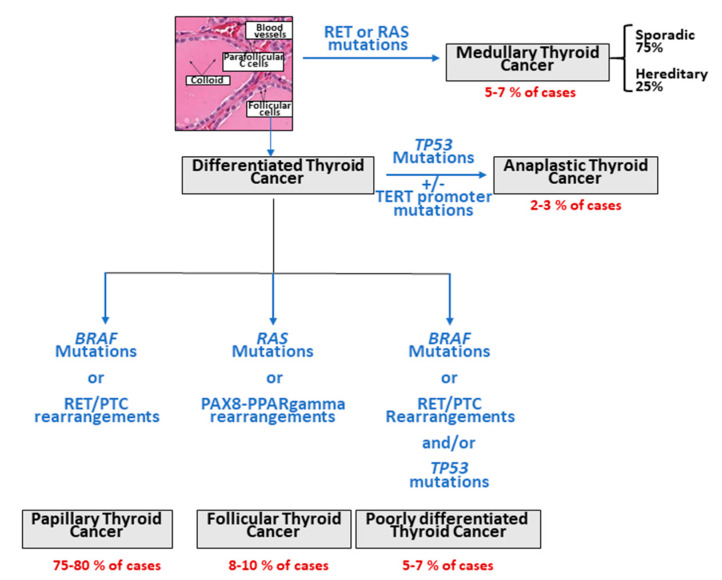
Different histological types of thyroid carcinomas and most relevant/driver molecular alterations.

Thyroid carcinoma is the most frequently reported endocrine neoplasia and represents 3–4% of all human tumors. Accordingly to their histological features, different types of thyroid carcinoma are described: well-differentiated papillary thyroid carcinoma (PTC, 75–80%), poorly differentiated thyroid carcinoma (PDTC, 5–7%), follicular thyroid carcinoma (FTC, 8–10%), anaplastic thyroid carcinoma (ATC, 2–3%), which originate from follicular cells, and medullary thyroid carcinoma (MTC, 5–7%), which derives from parafollicular C-cells (Figure 1) [1]. PTC, FTC and ATC are very rarely familial (only about 5% of PTC patients), while MTC is inherited as an autosomal dominant trait in 25% of cases.

Hereditary MTC can occur as an isolated form, familial MTC (FMTC) in which only tumors of the thyroid gland are present, or in association with neoplasia of other endocrine organs (i.e., parathyroid and adrenal glands), thus giving rise to the multiple endocrine neoplasia type 2 (MEN 2) syndromes. MEN 2 are then distinguished into two different subtypes (i.e., MEN 2A, MEN 2B) according to the different clinical manifestations [2].

In the last decades, many studies have been performed to find the genetic alterations involved in the pathogenesis of thyroid cancer. The first studies were mainly based on the analysis of one or a few genes, and only a limited number of gene alterations were investigated. Finally, after the improvement of sequencing techniques (next-generation sequencing, NGS) that are nowadays able to investigate large portions of the genome and even the whole-genome, exome or transcriptome or any other “home” with a quite easy approach, the genomic landscape of the different histotypes of thyroid cancer has been deciphered [3,4,5,6]. The aim of this review is to describe genetic markers relevant to thyroid cancer.

## 2. Oncogenic Alterations in PTC, FTC, PDTC and ATC

The great majority of the alterations involved in the pathogenesis of these tumors are represented by somatic mutations that likely occur in the early steps of the tumoral transformation process. The most frequent driver events can be either point mutations or gene rearrangements, mainly affecting the MAPK pathway and phosphatidylinositol-3 kinase (PI3K)/AKT pathway. The mutated genes that affect these pathways encode cell-membrane receptors with tyrosine kinases activity such as *RET* and NTRK1 and intracellular signal transducers, among which *BRAF* and *RAS*. On the other hand, PI3K/AKT pathway, which is mainly involved in FTC initiation, is driven by activating mutations in *RAS*, *PIK3CA*, and *AKT1* as well as by inactivation of *PTEN*. Mutations of *TP53* and Wnt/βcatenin are indeed involved in the progression from PTC to PDTC and ATC. Other altered genes, such as *TERT*, a ribonucleoprotein polymerase that maintains telomere ends, have been described in all the histological thyroid cancer types, with a significantly higher prevalence in aggressive and undifferentiated tumors, indicating their role in thyroid cancer progression.

Here following we will discuss the major players in the process able to transform a normal follicular cell into a malignant cell and to determine tumoral proliferation.

### 2.1. RET Rearrangements

**Figure 2:**
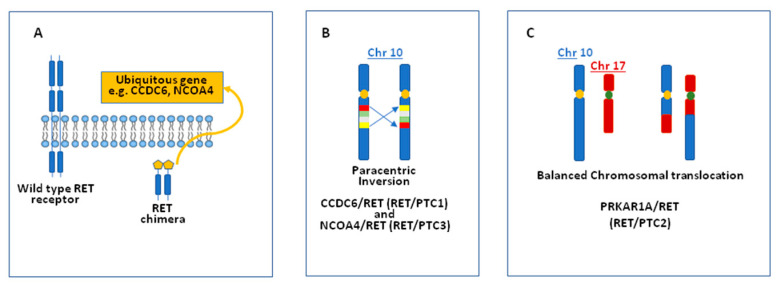
Mechanisms of RET/papillary thyroid carcinoma (PTC) activation. (**A**): the tyrosine kinase domain of the RET gene is constitutively activated by the fusion with a ubiquitous gene; (**B**): a paracentric inversion on chromosome 10 leads to the formation of a RET chimera as RET/PTC1 and RET/PTC3; (**C**): a chromosomal translocation leads to the formation of a RET chimera as RET/PTC2.

The first rearrangement of the RET gene (named RET/PTC1) was described several years ago using DNA transfection analysis on NIH3T3 cells [7]. As a result of this rearrangement, the tyrosine kinase domain of the RET gene is fused to the promoter region of the CCDC6 gene (formerly called H4) that drives the ligand-independent activation of the RET/PTC protein. Soon after the first description of the RET/PTC1 other RET fusions have been discovered. In particular, the RET/PTC3 rearrangement, in which RET is fused to the promoter region of the NCOA4 gene (also known as ELE1), was described in a post-Chernobyl thyroid tumor [8]. Over the years, several RET/PTC rearrangements have been reported in thyroid carcinoma, all characterized by the fusion of the tyrosine kinase domain of the receptor with a ubiquitous driver gene that allows the illegitimate kinase expression in cells that commonly do not express it (i.e., follicular cells) (Figure 2A). The gene rearrangements can be due to either a paracentric intrachromosomal 10 inversion or a translocation between chromosome 10 and another one (Figure 2B,C).

In most cases, these rearrangements differ for the involvement of different partner genes, but in few cases, different RET/PTC rearrangements are characterized by the occurrence of different breakpoints giving origin to longer or shorter chimeras [9]. The high prevalence of RET/PTC in post-Chernobyl thyroid tumors (87%) highlighted a strong relation between RET rearrangements and radiation exposure [10,11]. Years later, it has been proposed by in vitro experiments that the spatial proximity of the loci involved in RET/PTC rearrangements predisposes their mis-joining as a consequence of double-stranded breaks produced by ionizing radiation [12,13,14,15].

RET/PTC rearrangements have been found at a higher frequency in radiation-exposed children than in adults (probably due to the high proliferation rate of thyroid follicular cells in childhood) [10,11,16]. It is a matter of fact that with the increase of the latency period from the nuclear accident, the prevalence of RET/PTC rearrangements declined [17]. Interestingly a statistically significant decrease in the RET/PTC prevalence also has been reported by some authors in sporadic PTC [18]. A relatively low prevalence of RET/PTC rearrangements (6.3%) has been reported when using a next-generation sequencing approach [3]. Although to a much lower extent, rearrangements of the RET gene have also been reported in benign nodules as well as in Hashimoto thyroiditis [10,19,20].

The prognostic value of RET/PTC rearrangement in thyroid cancer has not been fully clarified yet. In a consecutive series of 1510 patients with thyroid cancer, RET/PTC-positive cases tended to be more aggressive with respect to RAS-positive cases [21].

In addition, among PTC tumors with a RET rearrangement, RET/PTC1 was found to be associated with a small, classic PTC variant [22]. At variance, RET/PTC3 rearrangement is prevalent in the solid variant of PTC and with a more aggressive clinical presentation both in post-Chernobyl childhood thyroid cancer [23] and in sporadic cases [24]. A low prevalence of RET rearrangements, mainly in carcinoma associated with a differentiated component, have been found in PDTC and ATC [25].

### 2.2. Other Rearrangements

The possibility to use sequencing techniques characterized by a very high sensitivity has allowed the identification of additional rearrangements, other than of RET, in thyroid cancer, mainly in radiation-induced tumors. Although gene rearrangements involving BRAF oncogene were previously described by Ciampi et al. [26] in a series of radiation-induced post-Chernobyl thyroid cancer, they have also been reported in the TGCA study in sporadic cases [3]. In particular, a total of 13/484 (2.7%) fusion of the BRAF gene with different gene partners were identified (Table 1). Very few cases (4/484 (0.8%)) of PAX8/PPARgamma fusions were also found in the TCGA series, in particular in the follicular variant of PTC. Conversely, PAX8/PPARgamma fusions are much more frequent in FTC, being present in about one-third of them and with a prevalence ranging from 12 to 56% [27,28].

Although to a less extent, translocations were also found in other genes such as NTRK (1.2%), THADA (1.2%), ALK (0.8%) and FGFR2 (0.4%) [3]. In addition to PTC, ALK fusions were found with a high prevalence in PDTC and ATC [29]. A high prevalence (55.5%) of less common gene fusions (STRN/ALK, TPR/NTRK1, SQSTM1/NTRK3, AFAP1L2/RET, and PPFIBP2/RET) (*n* = 5) were also reported in Fukushima PTC previously found to be negative (*n* = 9) for the classical oncogenes, thus confirming the strong correlation between radiation exposure and gene translocations [30].

### 2.3. BRAF Point Mutations

The BRAF gene encodes for a protein belonging to the serine/threonine-protein kinase family. This protein plays a role in regulating the MAP kinase/ERKs signaling pathway, which affects cell division, differentiation, and secretion. Mutations in this gene are associated with various human cancers, including non-Hodgkin lymphoma, colorectal cancer, malignant melanoma, thyroid carcinoma, non-small cell lung carcinoma, and adenocarcinoma of the lung. The most common mechanism of BRAF activation is the c.1799T > A, p.V600E (COSM476) point mutation that, according to the Cosmic database (https://cancer.sanger.ac.uk/cosmic), accounts for 51% of PTCs. A similar rate of BRAF mutations in PTC (59.7%) also has been reported by the Cancer Genome Atlas Research (TGCA) study [3] that was performed by NGS on a series of about 500 PTC cases. All point mutations and the rearrangements lead to the activation of BRAF kinase and to the chronic stimulation of the MAPK pathway.

The BRAF-V600E mutation constitutes 98–99% of all BRAF mutations found in thyroid cancer, but other alterations, including other point mutations, in-frame insertion/deletion and rearrangements, have been reported (Table 2). However, BRAF “rare” mutations are mainly present in the follicular variant of PTC and correlate with a good outcome [29]. Interestingly BRAF amplifications have been frequently found in BRAF wild-type tumors [30]

A different prevalence of BRAF mutations has been observed according to different morphological variants of PTC, and the highest prevalence was found in the tall cell PTC than in the classic variant, while a rather low prevalence has been reported in the follicular variant [21], where also some rare BRAF mutations are found [29].

Although not confirmed in an American series [31], a statistically significant increase in the BRAF mutation prevalence in sporadic PTC cases has been reported in parallel with the abovementioned decrease in RET rearrangement prevalence [18,32,33]. Since it has been demonstrated that high iodine levels could be a risk factor for BRAF mutations [34], the increased occurrence of BRAF mutations in PTC has been hypothesized, although never demonstrated, to be related to the intake of prophylactic iodine that has become increasingly recommended. Another hypothesis is that exposure to pollutants can be responsible for the induction of BRAF-V600E mutation, and in particular, a correlation has been found between a higher prevalence of BRAF-V600E mutation and the living close to the Etna volcano [35].

BRAF mutations have been shown not to be a major event in post-Chernobyl thyroid carcinomas as well in a not irradiated pediatric population, while these alterations have been found to be highly prevalent in thyroid carcinomas in the young population of Fukushima, suggesting a different mechanism of the tumoral transformation in the 2 groups [36]. The BRAF-V600E mutation has also been demonstrated to be subclonal or even oligoclonal with a different technical approach [36,37,38,39]. These findings led to hypothesize that this mutation might not always be the first transforming genetic event but rather a secondary event in PTC tumorigenesis [40,41]. Nevertheless, a high percentage of BRAF-V600E mutated alleles, that indicates a clonal origin and development of tumoral cells, correlated with a specific PTC molecular subtype and predicts a poorer disease outcome [36].

Several studies have investigated the role of BRAF-V600E mutations as prognostic markers and have shown a strong correlation with poor clinicopathological outcomes of PTC. In particular, a close association of BRAF mutation with extrathyroidal extension, lymph node metastasis, and advanced TNM stages III/IV of PTC, which are well-documented risk factors associated with increased rates of recurrence and mortality of thyroid cancer, have been reported [42,43,44,45,46]. The role of the BRAF-V600E mutation as a poor prognostic factor has also been reported in low-risk intrathyroidal tumors [44]. Despite the above-reported studies, the association between the BRAF-V600E mutations with increased tumor aggressiveness and poor prognosis in PTC is still under debate [47,48]. At variance, PTC cases with rare BRAF mutations, and in particular the BRAF-K601E, show an indolent behavior similar to the cases with BRAF wild-type [29,49].

The BRAF-V600E mutation also occurs in about 30% of PDTC and 40% of ATC [4,50,51]. It is interesting to note that many of these carcinomas are characterized by the presence of well-differentiated areas, and BRAF-V600E is present in both tumor components assuming the hypothesis that this mutation is an early event in tumor development and dedifferentiation [52].

### 2.4. TERT Mutations

**Figure 3:**
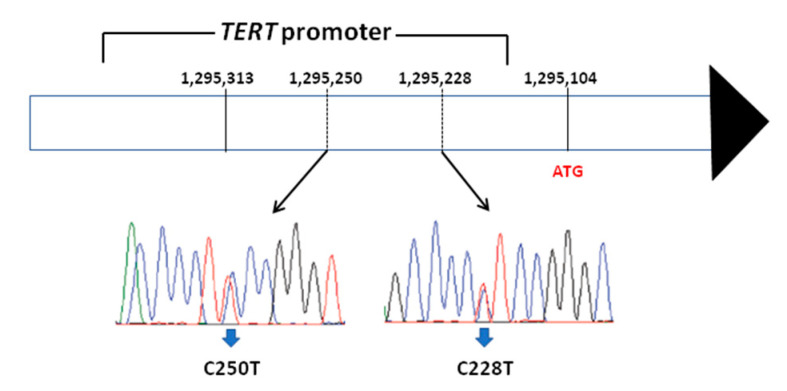
Schematic representation of TERT gene with the indication of the 2 most frequent mutations localized in the gene promoter, responsible for the transcription of the gene starting from the ATG codon.

Telomerase reverse transcriptase (TERT) is the catalytic domain of telomerase whose role is to add telomeres, to preserve chromosomal integrity and genome stability [53,54]. In addition, TERT has been shown to play a major role in the activation of telomerase during malignant transformation of cells [55,56]. In 2013, using whole-genome sequencing, mutations in the promoter of the TERT gene have been described in melanoma [57,58] and in other human cancers, among which thyroid cancer [59]. The two most common TERT promoter mutations occurring in thyroid cancer are located in the promoter region (chr: 5, 1,295,228 C > T (C228T) and 1,295,250 C > T (C250T)) (Figure 3).

These two mutations are mutually exclusive, suggesting that both the two mutations are highly transforming. The most frequent TERT mutation in thyroid carcinoma is the C228T mutation, and its prevalence is increasing with the increase of the level of aggressiveness being lower in the less aggressive PTC and very high in the more aggressive ATC. A similar association of TERT mutations and aggressiveness of the disease is also observed when comparing different histological variants of PTC. Pediatric thyroid tumors, which are considered not so aggressive, have been found to be negative for the presence of TERT promoter mutations [58]. To date, no TERT promoter mutations have been found in benign thyroid diseases and in MTC [59,60].

TERT promoter mutations have been found to correlate strictly with poor clinicopathological features and bad outcomes of the tumor [60]. A significant association between TERT promoter mutations with older age at diagnosis, tumor size, extrathyroidal invasion, vascular invasion, lymph node and distant metastasis, advanced stage and mortality has been reported [61]. TERT promoter mutations have been found to be associated with the presence of the BRAF-V600E mutations: the coexistence of BRAF-V600E and TERT mutation has been demonstrated to define a particularly aggressive group of thyroid tumors and in particular with tumor recurrence and mortality [60].

### 2.5. RAS Mutations

**Figure 4:**
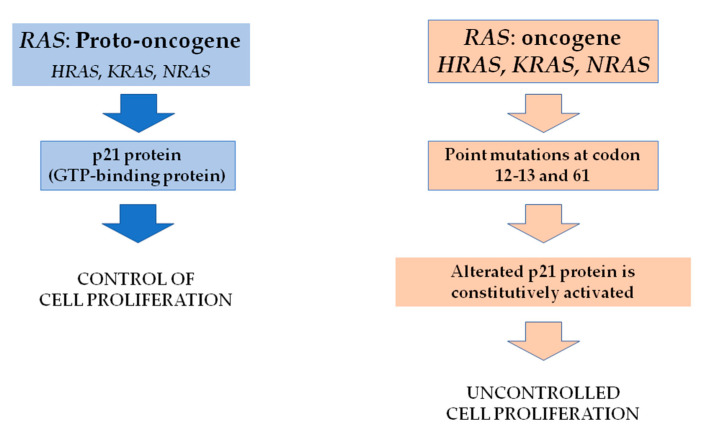
RAS gene activation in cancer: The RAS oncogene encodes for the p21 protein. In its native state, p21 controls cell growth and differentiation. When a point mutation occurs at codons 12, 13 and 61, p21 is constitutively activated, leading to uncontrolled cell growth.

Proteins of the RAS family are G-proteins able to activate the MAPK and other signaling pathways. In their active state, RAS proteins bind GTP; when GTP is hydrolyzed to GDP, ras proteins assume their inactive state (Figure 4).

Three isoforms of the RAS gene exist: H-RAS, K-RAS and N-RAS being N-RAS the most mutated in differentiated thyroid tumors, mainly at codon 12, 13 and 61 and H-RAS, K-RAS in MTC. According to the data of the TGCA study that published the results of a comprehensive next-generation study on PTC [3], the overall prevalence of N-RAS, H-RAS and K-RAS was 8.5%, 3.5% and 1%, respectively, very similar to that derived from the COSMIC database. Interestingly all RAS mutations reported by the TGCA study are in the follicular variant of PTC; thus, the relative frequency of the mutations in this subgroup is really higher in keeping with the evidence that RAS mutations are a major leading event in FTC [62]. Based on the presence of BRAF or RAS mutations, two main classes of PTC have been identified and have been named “BRAF-like and RAS-like” [3,62]. In detail, the first group of tumors was mainly constituted by classical or tall cell variants and with a significant reduction of the expression of the thyroid differentiation genes. At variance, the RAS-like PTCs were mainly follicular variants characterized by a high degree of differentiation. Nevertheless, RAS mutations have been found to be particularly relevant also in poorly differentiated and anaplastic thyroid cancer, where are frequently associated with other mutations such as TERT promoter mutation [63]. Somatic mutations in the RAS family have been reported in FTC. N-RAS mutations at codon 61 have been found to be mutated at a prevalence varying from 15% up to 40% of FTC [64,65]. New insights from a single-center and a large patient cohort of RAS mutated FTC have been shown to increase the metastatic potential and disease-specific mortality.

### 2.6. EIF1AX Mutations

The TGCA study [3] identified EIF1AX as a novel cancer gene in PTC. Mutations of EIF1AX were found in 1.5% of cases. The EIF1AX gene encodes for a eukaryotic translation initiator factor involved in the control of the initiation of protein synthesis. Exons 2, 5 and 6 of the EIF1AX gene have been further analyzed in other studies [66] and 3/86 (2.3%) PTC, 1/4 (25%) ATC and 2/27 (7.4%) follicular adenomas were found to carry one of these somatic mutations. The important role of the EIF1AX gene in thyroid tumorigenesis came up also by the NGS studies on PDTC, ATC and FTC in which an EIF1AX gene mutation was found in 11%, 9%–13% and 5.1% of cases, respectively [4,51,65]. According to the data of the TGCA study on PTC, the EIF1AX mutations result to be mutually exclusive with any other mutation. At variance, EIF1AX mutations seem to co-occur with RAS mutations in more advanced tumors.

### 2.7. TP53 Mutations

**Figure 5:**
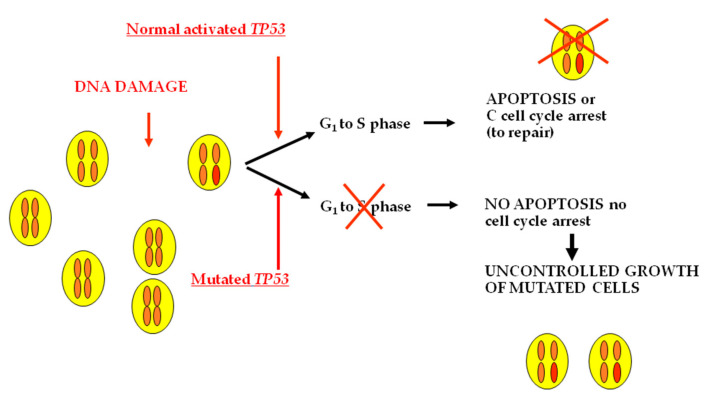
TP53 gene encodes for a protein involved in the control of the cell cycle. When DNA damage occurs, TP53 is able to induce the arrest of the cell cycle, and mutated cells cannot give origin to altered clones. Mutated TP53 loses the ability to stop the growth of mutated cells. Thus, tumoral clones take over.

The TP53 gene-encoded protein is involved in several cellular processes. In response to cellular stress, it can induce cell cycle arrest, apoptosis, senescence, DNA repair, or changes in metabolism (Figure 5).

In thyroid cancer, mainly in ATC, TP53 mutations are prevalently located in exons 5–9, and codon 273 is the most frequently involved [67,68]. The profile of TP53 mutations has been changing over the years mainly because the recent development of the highly sensitive NGS has allowed the identification of genetic alterations that are present at very low prevalence. By Sanger sequencing inactivating TP53 mutations were reported in about 26% of PDTC [69] and in about 80% of ATC. No TP53 expression or mutations were found in normal thyroid or in benign lesions [70]. According to the data reported by the TGCA study, TP53 mutations are present in a negligible percentage (0.7%) of PTC. At variance, using a last-generation sequencing approach [4,50,51], the prevalence of TP53 mutations is rather elevated in PDTC and ATC. The overall prevalence of inactivating TP53 mutations in ATC, which varied slightly in the aforementioned studies, is approximately 58% [63]. TP53 mutations can be found in ATC either associated with other genetic alterations typical of PTC or FTC, thus suggesting that ATC can derive from the dedifferentiation of well-differentiated longstanding thyroid cancer or as a unique genetic event, thus suggesting a direct role of these mutations in transforming the normal follicular cell into an undifferentiated tumoral cell. In this regard, it is worth noting that Landa et al. showed that none of the nine patients with TP53-positive ATC had any mutation in other components of the MAPK pathway, supporting the hypothesis that ATC can directly develop from follicular cells. This hypothesis is also supported by the observation that when a tumor is composed of a mixture of well-differentiated and undifferentiated areas, TP53 mutations are restricted to the undifferentiated areas of the tumor. TP53 mutations can also be found in PDTC, but their prevalence is significantly lower with respect to that found in ATC (73% vs. 8%) [4]. Moreover, in series in which the PDTC identification was done according to the Turin classification, TP53 mutations were demonstrated to be highly prevalent in ATC but completely absent in PDTC, suggesting a different genetic origin of the two malignancies [71]. A few prevalences of TP53 mutations have also been reported in FTC [65]

## 3. Oncogenic Alterations in MTC

The genetic landscape of medullary thyroid cancer (MTC) is not yet fully discovered, and about 40% of sporadic MTC and 2% of hereditary cases are still orphans of driver mutations. At present, mutations in the RET gene, both somatic and germline, appear to be the most important genetic events in MTC [9,72]. With the exception of the RAS gene, very few alternative gene alterations have been described in MTC [5,6,73]. As an alternative to point mutations, ALK and RET rearrangements have also been reported in few cases [73,74].

Here following we will discuss the major players in the process able to transform a normal parafollicular C-cell into a malignant cell and give origin to MTC.

### 3.1. RET Mutations

**Figure 6:**
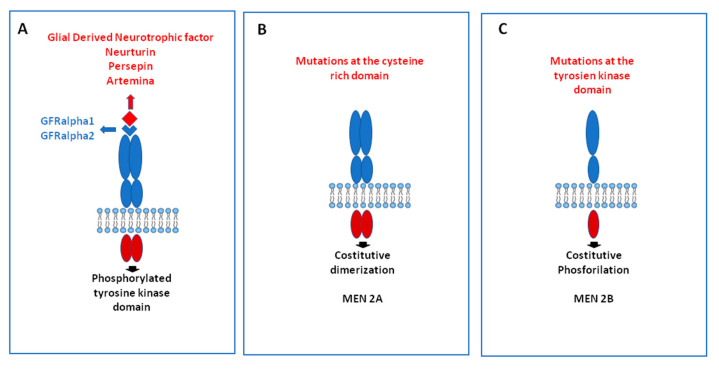
Mechanisms of activation of the RET gene: in physiological conditions, the binding of a RET ligand, which is mediated by a co-receptor, induces the dimerization of two to RET molecules, causing the phosphorylation of the tyrosine kinase domain (**A**). When a point mutation in the cysteine domain is present, as it happens in multiple endocrine neoplasia (MEN) type 2A syndrome, the constitutive dimerization of 2 RET molecules occurs, and the receptor is activated independently by ligand-binding (**B**). Alternatively, if the mutation occurs in the tyrosine kinase domain, as it happens in MEN 2B syndrome, constitutive phosphorylation activates the RET receptor independently by ligand-binding (**C**).

The RET proto-oncogene encodes for a tyrosine kinase transmembrane receptor whose ligands are members of the glial-derived neurotrophic factor family. Following the binding with the ligands, two RET receptor molecules make a dimer and initiate the activation of the receptor. When point mutations occur, ligand-independent activation of the receptor takes place (Figure 6).

Activation of RET stimulates multiple downstream pathways such as the mitogen-activated protein kinase (MAPK), the phosphoinositide 3-kinase (PI3K). Activating RET point mutations in MTC were first described in 1993 [75]. Since that time, many studies have been performed in hereditary and sporadic cases demonstrating the oncogenic driver role of RET mutations in MTC.

*Hereditary cases:* RET germline mutations have been found in more than 98% of MEN 2 kindreds, and only a few families affected by hereditary MTC are “orphans” of germline mutations [9]. In addition to RET mutations, recently, a germline ESR2 mutation has been identified in a family as a novel cause of familial MTC/CCH and provides important insights into a novel mechanism causing increased RET expression in tumorigenesis [76]. However, so far, it appears that this germline ESR2 mutation is a “private” mutation of that specific family since no other kindreds carrying the same germline mutation have been described [77].

The causative role of germline RET mutations in MEN 2 syndromes and the strict correlation between genotype and phenotype was clearly demonstrated by the study of the International RET Consortium that collected and published very important data about the RET mutations and the clinical and pathological features of 477 kindred affected by MEN 2A, MEN 2B and FMTC [78]. Following this study, several MTC series have been reported and summarized in reviews and or guidelines. One of the most important observation is that the classical MEN 2A phenotype is mainly associated with mutations in the RET cysteine codons 609, 611, 618, and 620 in exon 10 and, mostly, with the C634R mutation in exon 11. Secondly, MEN 2B was almost exclusively associated with the M918T mutation in exon 16. Few MEN 2B families have been found to have the A883F mutations: in these families, MTC is less aggressive than the M918T MTC tumors. By contrast, in FMTC cases, mutations were distributed among different codons/exons of the RET gene [78], but they are mainly concentrated in non-cysteine codons, such as codon 804 in exon 14 and codons 883 and 891 in exon 15 [78,79]. Almost all mutations reported to date are listed in public databases (www.hgmd.cf.ac.uk; www.arup.utah.edu/database/MEN2; www.ensembl.org, accessed date: 30 November 2020) (Table 3).

As clearly reported in the ATA guidelines, not all mutations confer the same aggressiveness to MTC [80,81], and this aggressiveness is correlated with the transforming ability of the RET mutation. We and others demonstrated that M918T mutation and mutations ad codon 634 are more transforming than non-cysteine mutations [82]. Because of the pathogenic role of germline RET mutations and their correlation with phenotype, all cases of MTC, both those with a clear familial-positive history and those apparently sporadic, must be submitted to the RET genetic screening. This will allow the correct identification of the hereditary cases and of the gene carriers among their first-degree relatives. These latter will be studied for the presence of an undiagnosed but already present MTC or for their potentiality to develop the tumor if not yet present. The planning of a prophylactic surgical treatment or the follow-up strategy will be done according to the age of the patients, the type of RET mutation and the levels of serum calcitonin [83,84].

*Sporadic cases*: According to data collected from several studies and published in a public database (COSMIC, https://cancer.sanger.ac.uk/cosmic), RET somatic mutations have also been found in 932/2107 (44%) sporadic MTC tissues. The most frequent RET somatic alterations in sporadic MTC are point mutations, but deletions and insertions have also been reported. Although RET somatic mutations have been found at different codons, the M918T mutation in exon 16 is the most frequently reported, especially in more advanced cases [83,85]. The prevalence of RET somatic mutations in MTC was around 50% of cases in series in which direct sequencing was adopted for the analysis. With the introduction of advanced sequencing methodologies (next-generation sequencing, NGS) that allowed the deep sequencing of the larger portion of the genome, the role of somatic RET mutations as main drivers in MTC had been confirmed. As reported in a large series [86], RET mutations are almost always mutually exclusive, and only in few cases, multiple RET somatic mutations are present, suggesting that MTC is a rather stable tumor. This evidence was also reported in additional studies [87,88] that demonstrated that only in about 20% of cases a different RET mutation profile could be found when comparing primary tumor and its corresponding metastases. NGS studies have also allowed the definition of the frequency of the mutated allele (AF) and have demonstrated that larger tumors not only are characterized by a higher prevalence of RET somatic mutations [89] but also have a higher AF corresponding to a higher number of mutated cells. This evidence suggests the hypothesis that the presence of a RET mutation, particularly M918T, is able to induce a growth advantage resulting in the formation of larger and clonal tumors.

The presence of somatic RET mutations has been found to be correlated with a worse prognosis of the tumor and shorter survival [90,91]. The correlation between RET somatic mutation and a worse outcome is strengthened by the evidence that the prevalence of RET somatic mutations is higher in patients with a more advanced tumor and lower in patients with smaller tumors [85]. At variance with the RET genetic screening in the familial form, the search for somatic RET mutation in the tumoral tissue is not yet part of the routine clinical practice, although it is highly desirable to be new anti-RET-specific drugs under development.

### 3.2. RAS Mutations

Interestingly, in the last years, RAS mutations have also been found in MTC [86,92,93]. In 2011, a Portuguese group [92] found somatic HRAS and KRAS mutations in 15/26 (57.7%) and in 3/26 (11.5%) RET wild-type MTC cases. At variance, only 1/40 (2.5%) RET-positive case had a somatic RAS mutation, indicating that RAS and RET mutations are mutually exclusive in MTC. In the Ciampi et al. series [86], RAS mutations were present in 69.2% (18/26) of RET-negative cases and in only 2.5% of RET-positive sporadic MTC, confirming that activation of the RAS and RET proto-oncogenes represents alternative genetic events in sporadic MTC tumorigenesis. Although with different prevalence, likely due to technical and/or ethical reasons, these results have been confirmed by several groups. It is also worth noting that RAS mutation identifies a subgroup of MTC with less aggressive behavior when compared with cases with RET mutations [86].

## 4. Conclusions

In the last years, much progress has been made in deciphering the genetic landscape of thyroid carcinoma. In PTC, the results of the study of the TGCA study definitively demonstrated that genetic alterations are present in about 95% of tumors and that two main classes of tumors (BRAF- and RAS-like), each with their own clinical and biological behavior, can be distinguished. In addition to PTC, important achievements have also been reached for PDTC and ATC. BRAF and RAS mutations have been confirmed to play an important role in the pathogenesis of these tumors, and TP53 mutations have been found to be fundamental in tumor progression. It also has been clearly demonstrated that TERT promoter mutations and TP53 mutations are present with a high-frequency in more advanced tumors, frequently associated with other mutations, and their presence is a sign of aggressiveness. Similarly, the presence of several genetic alterations in the same tumoral tissue is correlated with a higher degree of dedifferentiation and probability of a bad outcome. As MTC are concerned, the whole-exome sequencing and target sequencing studies confirmed that mutations in the RET gene are the most common molecular events followed by H-RAS and K-RAS mutations. The comprehensive knowledge of the genetic events responsible for thyroid tumorigenesis, as well as for any other human tumor, is important to better predict the biological behavior and to better plan the therapeutic strategy for specific treatment of the malignancy on its molecular profile.

## Figures and Tables

**Table 1:** *BRAF* rearrangements in thyroid cancers.

Gene Fusion	Prevalence	Type of Cancer	Reference
*AKAP9/BRAF*		Radiation-induced	[26]
*SND1/BRAF*	3/33	Sporadic	[3]
*AGK/BRAF*	1/33	Sporadic	[3]
*AP3B1/BRAF*	1/33	Sporadic	[3]
*BLC2L11/BRAF*	1/33	Sporadic	[3]
*CCNY/BRAF*	1/33	Sporadic	[3]
*ERC1/BRAF*	1/33	Sporadic	[3]
*FAM114A2/BRAF*	1/33	Sporadic	[3]
*MACF1/BRAF*	1/33	Sporadic	[3]
*MKRN1/BRAF*	1/33	Sporadic	[3]
*SVOPL/BRAF*	1/33	Sporadic	[3]
*ZC3HAB1/BRAF*	1/33	Sporadic	[3]

**Table 2:** Rare BRAF mutation in thyroid cancers according to the COSMIC database.

Nucleotide Change	Amino Acid Substitution	Mutation Type	Prevalence
c.1834C > T	p.Q612	Substitution nonsense	2/40,072
c.1778G > A	p.G593D	Substitution missense	1/21,576
c.1793C > T	p.A598V	Substitution missense	2/21,576
c.1796C > G	p.T599R	Substitution missense	1/21,576
c.?	p.T599I	Substitution missense	3/21,576
c.1801A > G	p.K601E	Substitution missense	54/21,576
c.1794_1795insGTT	p.A598_T599insV	Insertion inframe	8/13
c.1795_1796insTAA	p.A598_T599insI	Insertion inframe	1/13
c.1795_1796ins27	p.A598_T599insKKIGDFGLA	Insertion inframe	1/13
c.1796_1797insTAC	p.T599_V600insT	Insertion inframe	1/13
c.1797_1798ins9	p.T599_V600insETT	Insertion inframe	1/13
c.1798_1799ins18	p.T599_V600insDFGLAT	Insertion inframe	1/13
c.?	p.K601del	Deletion inframe	3/6
p.V600_W604del	p.V600_W604del	Deletion inframe	1/6
c.1801_1803delAAA	p.K601del	Deletion inframe	1/6
c.1801_1812del12	p.K601_W604del	Deletion inframe	1/6
c.1799_1814 > ATGT	p.V600_S605 > DV	Complex	1/33
c.1796_1809 > TC	p.T599_R603 > I	Complex	4/33
c.1799_1801delTGA	p.V600_K601 > E	Complex	15/33
c.1798_1798G > TACA	p.V600 > YM	Complex	4/33
c.1796_1798CAG > TAGCTT	p.T599_V600 > IAL	Complex	2/33
c.?	p.T599_V600 > IYI	Complex	1/33
c.?	p.T599_R603 > I	Complex	1/33
c.?	p.V600_K601 > E	Complex	1/33
c.?	p.V600 > YM	Complex	1/33
c.1799_1801delTGA	p.V600_K601 > E	Complex	3/33

**Table 3:** Distribution of germline *RET* mutation in hereditary medullary thyroid carcinoma (MTC).

Location	Protein Change	Classification	MEN2 Phenotype
Exon 5	p.V292M	Pathogenic	MEN2A and FMTC
	p.T338I	Pathogenic	
Exon 7	p.505_506del	Pathogenic	MEN2A
Exon 8	p.C515S/W	Pathogenic	
	p.G533C	Pathogenic	
Exon 10	p.C609R/G/Y/S/F	Pathogenic	MEN2A and FMTC
	p.C611S/R/G/YF/W		
	p.C618S/R/G/Y/F/W		
	p.C620S/R/G/L/F/W/Y		
Exon 11	p.D631Y/A/G/V/E	Pathogenic/uncertain	MEN2A
	p.C634S/R/G/Y/L/W	Pathogenic	
	p.K666E/R	Pathogenic	
Exon 13	p.E768D	Pathogenic	MEN2A and FMTC
	p.L790F	Pathogenic	
Exon 14	p.V804M	Pathogenic	MEN2A and FMTC
	p.V804L	Pathogenic	
	p.Y806C	Benign	
Exon 15	p.A883T	Pathogenic	FMTC, MEN2B and MEN 2A
	p.A883F	Pathogenic	
	p.S891A	Pathogenic	
	p.S904F	Pathogenic	
Exon 16	p.M918T	Pathogenic	MEN2B and FMTC
	p.M918V	Pathogenic	
